# Recurrent pneumoperitoneum with pneumatosis cystoides intestinalis in tuberculous pleurisy: Case report

**DOI:** 10.1097/MD.0000000000046543

**Published:** 2025-12-12

**Authors:** Xiang Qiu, Lishan Wang, Muchu Daren

**Affiliations:** a Department of Radiology, Chengdu Integrated TCM&Western Medicine Hospital, Chengdu, China; b Department of Radiology, Dege County People’s Hospital, Dege, China.

**Keywords:** pneumatosis cystoides intestinalis, pneumoperitoneum, tuberculous pleurisy

## Abstract

**Rationale:**

Pneumatosis cystoides intestinalis (PCI) is a rare condition for which autopsy studies have reported a prevalence of 0.03% in the general population. Although PCI can be associated with pneumoperitoneum, many cases are benign and do not reflect viscus perforation. Coexistence with tuberculous pleurisy and recurrent pneumoperitoneum is rare and may be underrecognized.

**Patient concerns:**

In this report, we present the case of a 38-year-old Tibetan male who experienced tuberculous pleurisy accompanied by recurrent pneumoperitoneum with PCI. Despite the presence of free air, he remained minimally symptomatic. Comprehensive imaging failed to reveal evidence of intestinal perforation, ischemia, or necrosis. Consequently, a nonoperative management strategy was successfully employed, leading to the patient’s recovery and discharge.

**Diagnoses:**

The patient denied any clinical symptoms other than mild abdominal distension. Computed tomography demonstrated PCI involving the transverse colon and hepatic flexure with scattered intraperitoneal free air and no evidence of perforation, ischemia, or necrosis.

**Interventions:**

Nonoperative management comprised close hemodynamic and abdominal monitoring, bowel rest, low-flow oxygen, and scheduled computed tomography reexamination. Standard antituberculosis therapy was continued.

**Outcomes:**

In our case, recurrent benign pneumoperitoneum improved with conservative care alone, supporting a standardized, threshold-based, individualized nonoperative approach with ongoing surveillance. Clinically stable PCI patients with pneumoperitoneum and no radiologic or clinical signs of perforation, ischemia, or necrosis are best managed nonoperatively; surgery is reserved for high-risk features.

**Lessons:**

Pneumoperitoneum secondary to PCI is often benign, may not require surgical intervention, and can recur. Tuberculous pleurisy may contribute to PCI with pneumoperitoneum via altered thoracoabdominal pressures. Characteristic computed tomographic findings are instrumental in recognizing PCI and distinguishing it from other causes of pneumoperitoneum.

## 1. Introduction

Pneumatosis cystoides intestinalis (PCI) is a rare condition characterized by multiple gas-filled cysts located within the submucosal or serosal layers of the gastrointestinal tract.^[1]^ Although PCI predominantly affects middle-aged to older adults, the median age in a recent scoping review was 53 years, indicating that younger patients can be affected as well.^[1]^ Autopsy studies have reported a prevalence of 0.03% in the general population; however, the actual incidence remains uncertain.^[2]^ In rare instances, PCI can lead to benign pneumoperitoneum and should be considered in the differential diagnosis of clinically stable patients, given the potential for nonoperative management.^[3]^ Multiple etiological factors have been implicated in its pathogenesis, including Crohn disease, intestinal stenosis, ulcerative colitis, medication use, extraintestinal disorders, and chronic obstructive pulmonary disease. The pathogenesis of PCI is believed to involve increased intra-intestinal pressure and accumulation of gas produced by aerogenic bacteria.^[4]^ However, there is a lack of multicenter prospective clinical trials evaluating the factors that distinguish benign pneumoperitoneum from pathologic pneumoperitoneum and guide their management.

## 2. Case presentation

**Figure 1.**
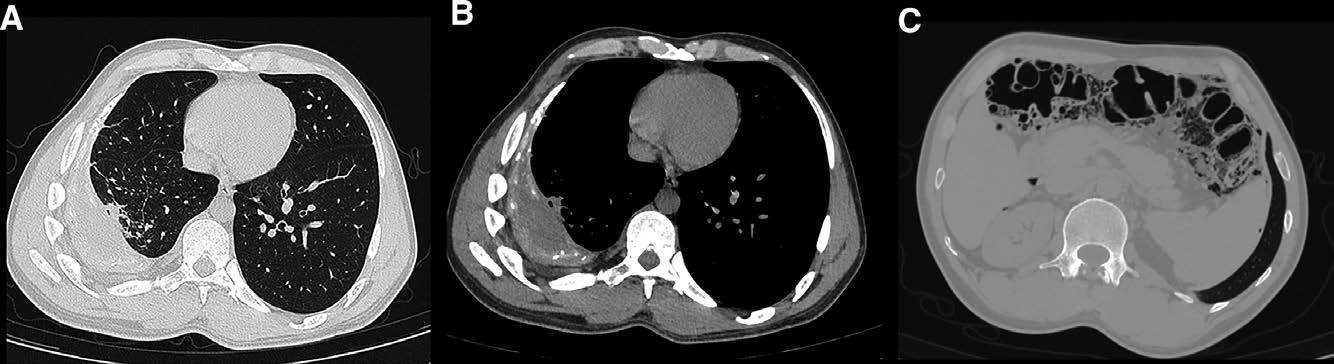
August 20, 2024, CT: (A) lung window and (B) mediastinum window revealed encapsulated pleural effusion in the right thoracic cavity, along with pleural thickening and calcification on the right side, (C) PCI with pneumoperitoneum. CT = computed tomograph, PCI = pneumatosis cystoides intestinalis.

**Figure 2.**
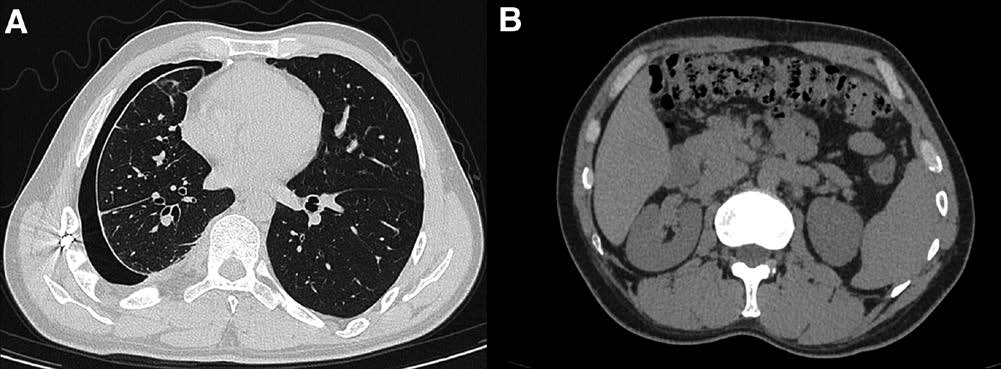
January 17, 2025, CT: (A) scan revealed a small amount of pneumothorax on the right side, (B) no significant pneumoperitoneum were observed in the abdominal cavity. CT = computed tomograph.

**Figure 3.**
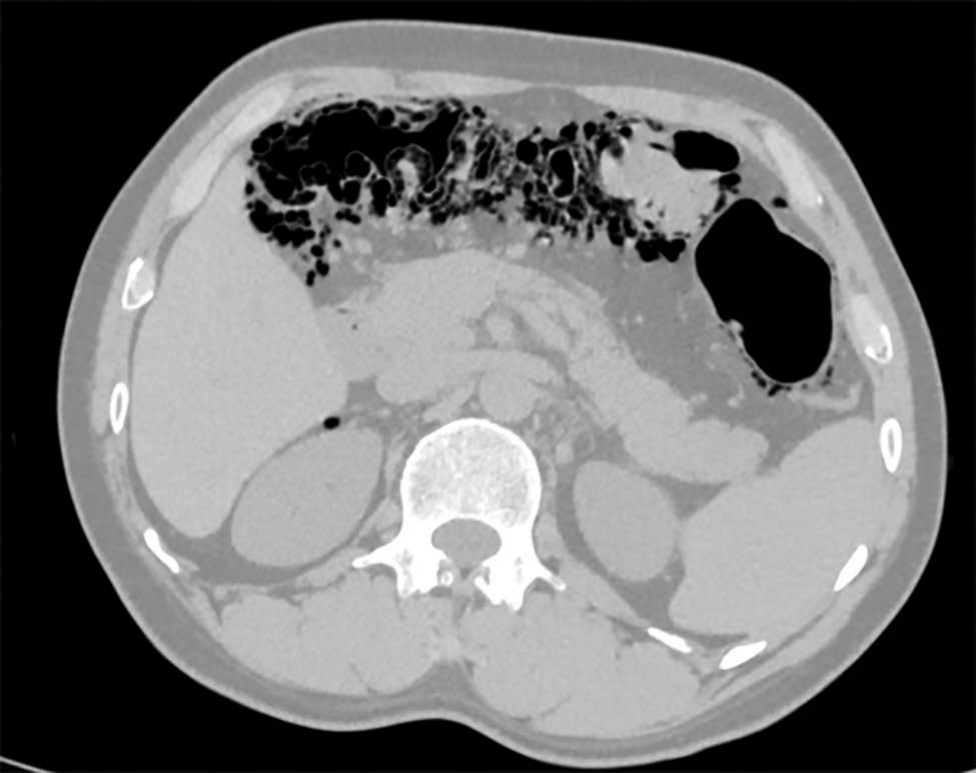
June 2, 2025, CT revealed PCI with pneumoperitoneum. CT = computed tomograph, PCI = pneumatosis cystoides intestinalis.

A 38-year-old Tibetan male presented with a 1-year history of cough, sputum production, and chest and back pain. On August 20, 2024, he was diagnosed with secondary pulmonary tuberculosis at a local hospital, and computed tomography (CT) revealed an encapsulated pleural effusion in the right thoracic cavity with pleural thickening, calcification, and cord-like and patchy opacities in the right lung (Fig. 1A and B). Additionally, PCI with pneumoperitoneum was also performed (Fig. 1C). On September 9, 2024, the patient underwent right thoracic pus drainage, pleural debridement, and lung repair under general anesthesia. The pathological findings were consistent with tuberculosis. On January 17, 2025, a follow-up CT was performed as part of routine postoperative surveillance after thoracic pus drainage, pleural debridement, and lung repair for tuberculous pleurisy. The patient reported intermittent mild chest discomfort, but no dyspnea, abdominal pain, fever, or peritoneal signs. The CT revealed a small right-sided pneumothorax. No significant PCI or pneumoperitoneum was observed in the abdominal cavity (Fig. 2A and B). Given the patient’s stable vital signs and minimal symptoms, we opted for conservative management with close monitoring of vital signs, supplemental oxygen as needed, and scheduled CT reexamination. On June 2, 2025 (approximately 6 months after thoracoscopy for tuberculous pleuritis), a follow-up chest CT was performed to assess postoperative recovery, including the resolution of the prior pneumothorax. Chest CT incidentally revealed free intraperitoneal air. Upon further examination, the patient reported only mild abdominal distension. Vital signs were stable and physical examination revealed no peritoneal signs. A dedicated abdominal CT subsequently demonstrated PCI involving the transverse colon and hepatic flexure, with scattered intraperitoneal free air (Fig. 3). Given the absence of peritoneal signs and benign imaging appearance without evidence of bowel ischemia, obstruction, or necrosis, we pursued conservative management consisting of rest, close monitoring of vital signs, and low-flow oxygen via a nasal cannula, and no surgical intervention was undertaken. The patient remained clinically stable and was discharged. He was instructed to return promptly for the evaluation of abdominal pain, nausea, vomiting, abdominal distension, or cessation of flatus and stool. Surgical intervention would be reconsidered only if clear peritoneal signs developed or if CT suggested bowel ischemia, obstruction, or necrosis.

## 3. Discussions and conclusion

This case involved a patient with tuberculous pleurisy in whom PCI with intermittent recurring pneumoperitoneum was incidentally identified. The patient remained clinically stable and was managed conservatively throughout. This stable course suggests that PCI-associated pneumoperitoneum can be benign and, in clinically stable patients, does not necessitate surgical intervention. Clinicians frequently encounter patients with PCI and pneumoperitoneum who undergo unnecessary surgery because of misdiagnosis of gastrointestinal perforation. In China, an exceedingly high surgical resection rate of 40.6% reflects insufficient awareness and misdiagnosis of this condition, despite the fact that many cases of PCI with pneumoperitoneum can recover with nonoperative management.^[5]^

PCI was classified into primary (15%) and secondary (85%) forms. Secondary PCI is associated with underlying conditions such as gastrointestinal obstruction, chronic obstructive pulmonary disease, abdominal trauma or surgery, and malnutrition. In addition to gastrointestinal disorders and emphysema, PCI has also been linked to rarer conditions, including the use of alpha-glucosidase inhibitors, sunitinib therapy, lung and bone marrow transplantation, systemic lupus erythematosus, systemic sclerosis, multiple myeloma, and granulomatosis with polyangiitis.^[4]^ According to Morris et al,^[6]^ 46% of the lesions occur in the colon, 27% in the small intestine, 7% in both, and 5% in the stomach. Approximately 3% of PCI patients develop complications such as pneumoperitoneum, volvulus, intestinal obstruction, or ischemia.^[4]^ In our case, PCI with pneumoperitoneum was a secondary complication of tuberculous pleurisy. Our patient was younger than the median age at PCI (53 years) reported by Costa et al.^[1]^ We hypothesized that this may have resulted from altered intra-abdominal pressure due to tuberculous pleurisy, which caused rupture of the intestinal mucosa and subsequent gas infiltration into the bowel wall. Notably, the patient’s prior surgery may have also contributed to the recurrence of PCI with pneumoperitoneum.^[5]^

In general, the diagnosis of PCI is not difficult, as typical radiographic findings include grape-like clusters or honeycomb-shaped lucencies along the bowel wall.^[5]^ The diagnosis of PCI relies primarily on imaging modalities including colonoscopy, CT, radiography, and ultrasonography.^[4]^ CT is considered the gold standard for confirming PCI, offering high diagnostic accuracy and the ability to detect other abdominal abnormalities.^[5]^ On CT, typical signs of acute intestinal disease or perforation, such as bowel wall discontinuity, segmental wall thickening, indistinct perivisceral fat planes, or abscess formation, warrant surgical intervention in PCI with pneumoperitoneum. However, most cases of PCI with pneumoperitoneum lack CT features suggestive of peritonitis, and ascites is uncommon; these are characteristic imaging features of benign PCI-associated pneumoperitoneum.^[7]^ Most of the asymptomatic patients recovered without intervention. For symptomatic patients, conservative management, including gastrointestinal decompression, bowel rest, parenteral nutrition, and correction of electrolyte disturbances, is usually effective.^[5]^ Successful treatment of PCI with pneumoperitoneum using conservative approaches, including hyperbaric oxygen therapy, has been reported in the literature.^[3]^ Conservative treatment is appropriate for PCI owing to its benign etiologies, whereas urgent surgery is indicated when bowel perforation, ischemia, or necrosis are suspected.

This single case, retrospective report limits causal inference and generalizability due to the patient’s specific context (tuberculous pleurisy, postoperative status) and reliance on imaging without endoscopic or pathologic confirmation. The proposed link between PCI/recurrent pneumoperitoneum and altered thoracoabdominal pressures remains hypothetical, lacking physiologic measurements or standardized biomarkers. These gaps underscore the need for prospective, multicenter studies with defined imaging and clinical thresholds. In our case, the patient remained stable and improved with conservative therapy from August 2024 to June 2025, supporting a criteria-based, individualized approach rather than routine surgery. In short, for clinically stable patients with PCI and pneumoperitoneum who show no imaging or clinical evidence of perforation, ischemia, or necrosis, nonoperative management is appropriate; surgery should be reserved for the emergence of high-risk features. Consistent with this principle, our recurrent PCI case with benign pneumoperitoneum improved steadily under conservative care without surgery, supporting a standardized, threshold-based, and individualized nonoperative strategy with ongoing surveillance.

## Acknowledgments

We would like to express our gratitude to the patients for granting permission to use their clinical data for this study and for the publication of this research.

## Author contributions

**Data curation:** Muchu Daren.

**Writing – original draft:** Xiang Qiu.

**Writing – review & editing:** Lishan Wang.
